# Supplementary material for: Prognostic Relationship Between Human Papillomavirus Status and Overall Survival in Patients with Tongue Cancer
Source: Viruses. 2025 May 29;17(6):780. doi: 10.3390/v17060780 (PMC12197418; doi:10.3390/v17060780)

viruses

Supporting Information

Article

Prognostic Relationship Between Human Papillomavirus Status and Overall Survival in Patients with Tongue Cancer

Chayanit Kritpracha [1], Peesit Leelasawatsuk [1], Virat Kirtsreesakul [1], Pasawat Supanimitjaroenporn [1], Jarukit Tantipisit [2] and Manupol Tangthongkum [1,*]

[1] Department of Otolaryngology Head and Neck Surgery, Faculty of Medicine, Prince of Songkla University, Hat Yai, Songkhla 90110, Thailand; ckk.chayanit@gmail.com (C.K.); kvirat2002@hotmail.com (V.K.); ohm_ps1@hotmail.com (P.S.); tmanupol@gmail.com (M.T.)

[2] Department of Pathology, Faculty of Medicine, Prince of Songkla University, Hat Yai, Songkhla 90110, Thailand; medew.jarukit@gmail.com

Figure S1 Kaplan–Meier survival curves for overall survival (A), disease-specific survival (B), and recurrence-free survival (C) in patients with tongue cancer.

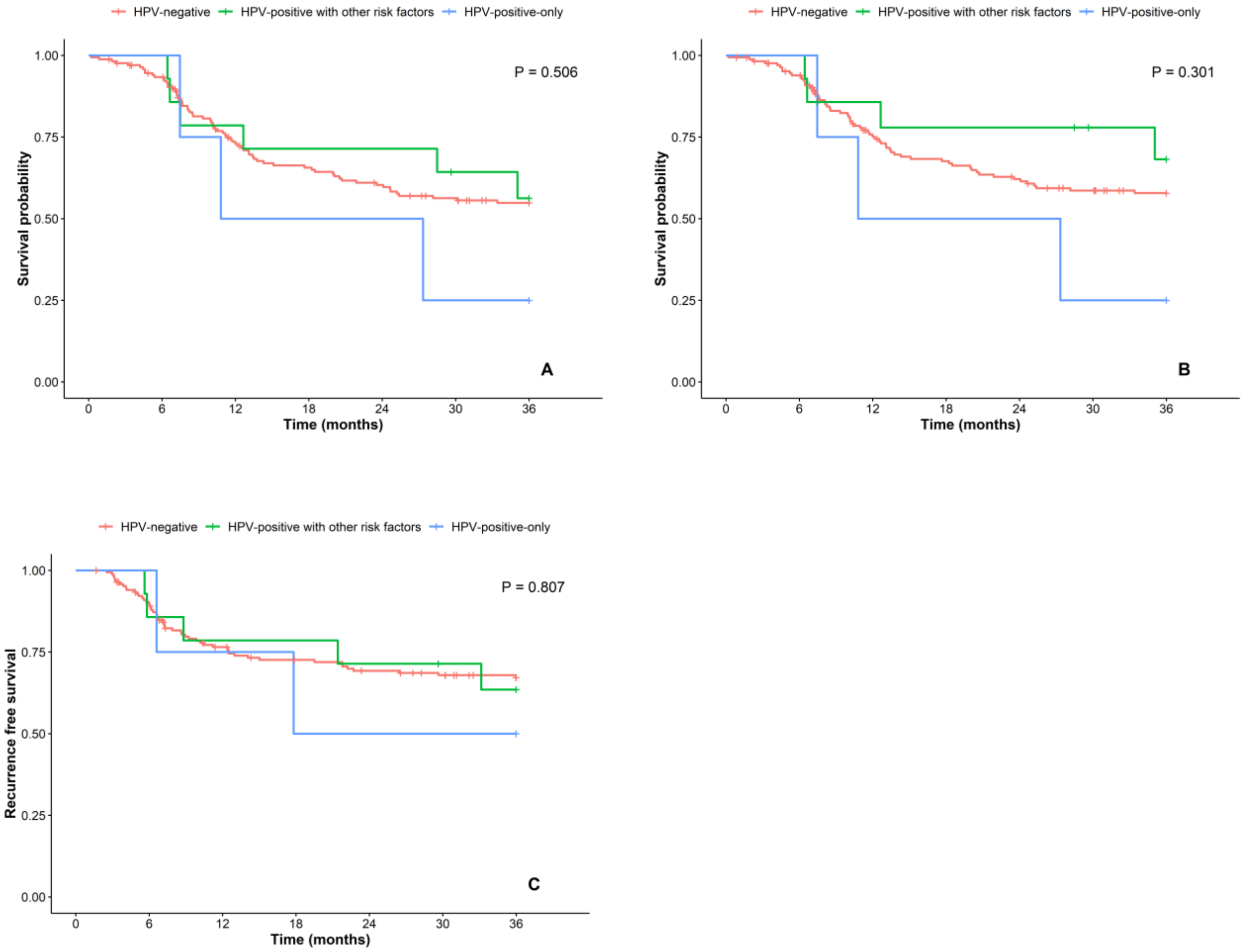

Supplement: Supplementary file 1 [file viruses-17-00780-s001.zip › viruses-3603719-supplementary.pdf]
